# The AI for Scientific Discovery Network^+^

**DOI:** 10.1016/j.patter.2020.100162

**Published:** 2021-01-08

**Authors:** Samantha Kanza, Colin Leonard Bird, Mahesan Niranjan, William McNeill, Jeremy Graham Frey

**Affiliations:** 1 School of Chemistry, University of Southampton, Southampton SO17 1BJ, UK; 2 School of Electronics and Computer Science and University of Southampton, Southampton SO17 1BJ, UK; 3 School of Humanities, University of Southampton, Southampton SO17 1BJ, UK

## Abstract

The Artificial Intelligence and Augmented Intelligence for Automated Investigation for Scientific Discovery Network^+^ (AI3SD) was established in response to the UK Engineering and Physical Sciences Research Council (EPSRC) late-2017 call for a Network^+^ to promote cutting-edge research in artificial intelligence to accelerate groundbreaking scientific discoveries. This article provides the philosophical, scientific, and technical underpinnings of the Network^+^, the history of the different domains represented in the Network^+^, and the specific focus of the Network^+^. The activities, collaborations, and research covered in the first year of the Network^+^ have highlighted the significant challenges in the chemistry and augmented and artificial intelligence space. These challenges are shaping the future directions of the Network^+^. The article concludes with a summary of the lessons learned in running this Network^+^ and introduces our plans for the future in a landscape redrawn by COVID-19, including rebranding into the AI 4 Scientific Discovery Network (www.ai4science.network).

## The Bigger Picture

The AI3SD Network^+^ was created to accelerate scientific discovery using new AAI techniques. Despite useful short-term impacts, this extensive space still has many unsolved challenges. It is vital that the trajectory of this effort continues, encouraging further collaborations, helping funding recipients realize their potential, and creating vital resources for the AAI for scientific discovery community.

A core aspect of our AI3SD philosophy is the importance of the human element in AI, hence why AAI for scientific discovery is our mantra. Humans must remain in the loop for any decision making that requires ethical consideration or the addition of human intelligence. The AI3SD approach combines the best of human and machine intelligence to gain notable acceleration in scientific discovery using transparent, responsible, and explainable AAI.

We aim to finish the first term of the AI3SD with a rich collection of resources and useful outputs, enabling members to progress toward the UN sustainability goals in the areas of chemicals and materials discovery. AI3SD will remain a sustainable entity only if members and organizers work together toward AAI.

## Graphical Abstract

**Figure:**
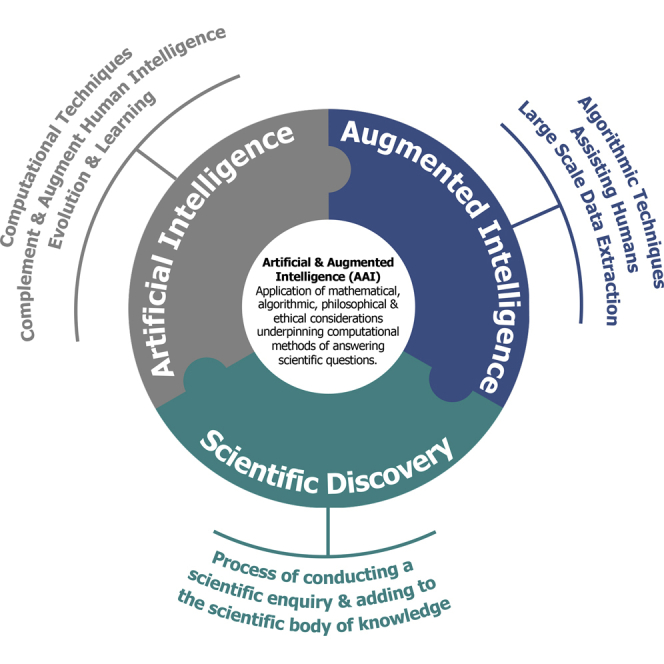

## Teaser

The Artificial Intelligence and Augmented Intelligence for Automated Investigation for Scientific Discovery Network^+^ has had an eventful first year, rapidly establishing a diverse community of different disciplines across academia and industry. We share with you our insights into the potential and challenges of the AI for scientific discovery space, coupled with what we have learned about running a research network.

## Introduction

In this perspective we describe the inception and first year of the Artificial Intelligence and Augmented Intelligence for Automated Investigation for Scientific Discovery Network^+^ (AI3SD^1^), funded by the Engineering and Physical Sciences Research Council (EPSRC^2^) of the UK Research Innovation (UKRI^3^) Network^+^.

In late 2017, EPSRC announced a call^4^ for a Network^+^ to promote groundbreaking research in artificial intelligence (AI) to accelerate cutting-edge scientific discovery. Our bid concentrated on chemical and materials discovery, as these are the areas with which we had familiarity and fields where it was apparent that major developments in industry and academia were already underway, offering huge potential to the community. We are sure that discoveries and techniques arising from our target areas will be transferable to the wider life sciences and beyond.

We were successful, were awarded the grant (EP/S000356/^5^), and were able in due course to appoint a very able network coordinator, which past experience in running the IT as a Utility (ITaaU) Network^+^^6^ had shown was an essential and difficult, multi-faceted role. Alongside this appointment, we also recruited a diverse interdisciplinary and extremely experienced advisory board from different academic and industrial institutions (the current members are all listed on our website^7^).

We note with some amusement that at the first Advisory Board meeting it was pointed out that we had missed the fact that if we had added a fourth AI then we could have become the AI4Science Network^+^. This highly convenient abbreviation inspired us to plan for a change in the Network^+^ name as we move the Network^+^ to a more self-sustaining future existence. While we retain the www.ai3sd.org URL, the http://www.AI4Science.Network will increasingly be used to point to the Network^+^ activities (further coincidences occurred because, on checking, we found that the URL www.ai4science.org was not available, and therefore obtained www.ai4science.network, although at a subsequent advisory board meeting we discovered that this www.ai4science.org was held by one of our own farsighted advisory board members who could not be present at the first advisory board meeting!).

**Figure 1:**
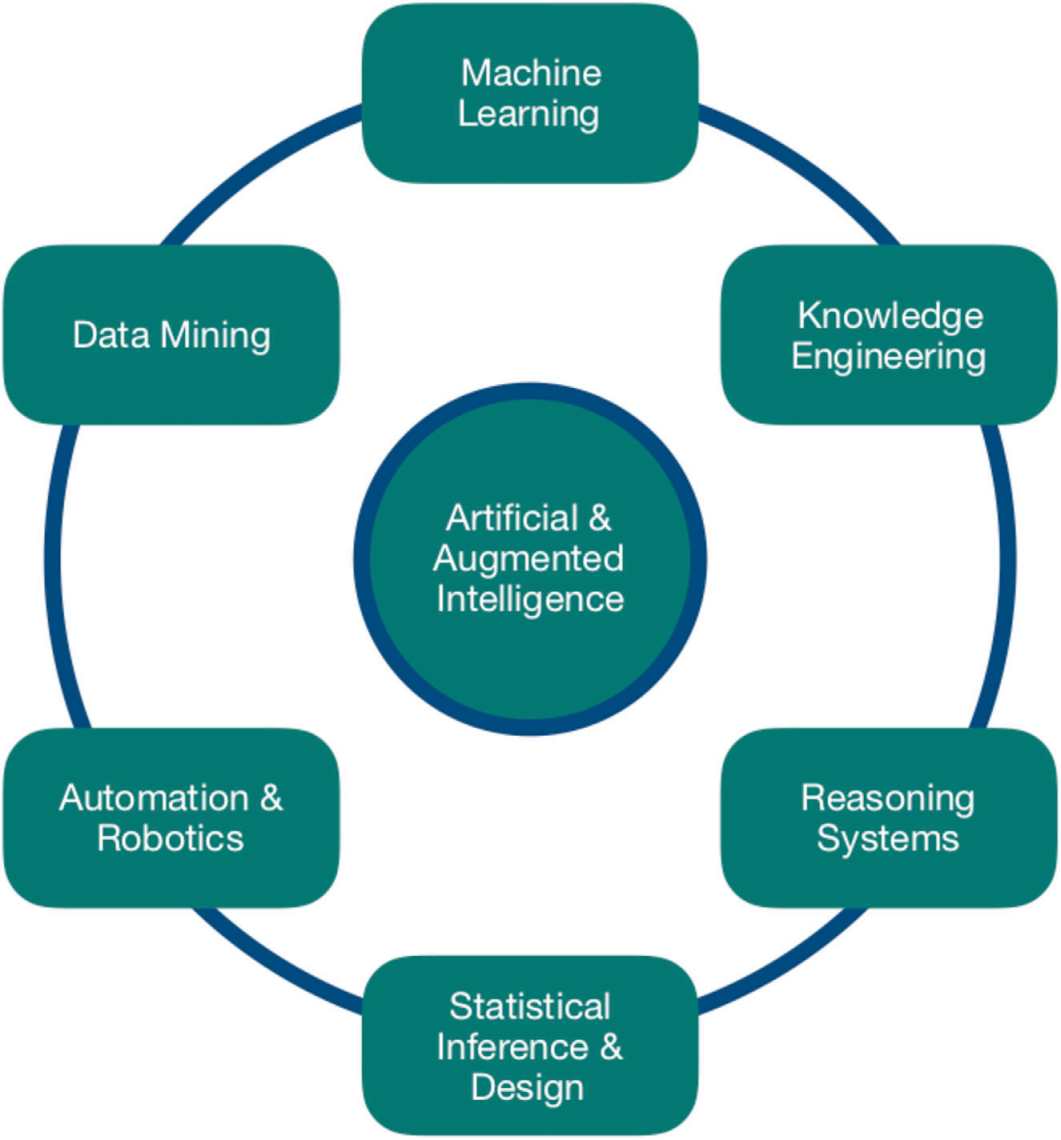
What is AAI? AAI is the outcome of applying a range of techniques that come from several diverse and traditionally quite different disciplines all brought together in our case to support an AI-enhanced problem-solving endeavor in the physical and life sciences.

The vision of the Network^+^ is centered on creating community-wide engagement at the cutting edge of both AI and scientific discovery, and we focused our activities not on simply applying known machine learning techniques to data-mining problems in the two problem domains but instead on growing a truly collaborative environment of multi-way interactions. Underlying this emphasis is the recognition that intelligent algorithms should have augmenting human intelligence as the goal (as described in Box 1 and Figure 1). These algorithms should exploit archived knowledge in their formulation and feed into higher-level symbolic representations, addressing the more philosophical issues of causality and uncertainty of complex reasoning systems. Such combined activity, when deployed, has to have ethical and explainable AI^8^^,^^9^ at its foundation.

Box 1 **Definitions**

In this article we refer to artificial and augmented intelligence (AAI). By AAI, we mean the application of mathematical, algorithmic, philosophical, and ethical considerations underpinning computational methods of answering scientific questions.

Artificial intelligence refers to computational techniques that complement and augment human intelligence, encompassing the notions of evolution and learning in the context of complex datasets.

Augmented intelligence refers to algorithmic techniques that assist in human problem solving, essentially by the extraction of useful information from large and complex datasets, which may be structured.

Scientific discovery is the process or product of conducting a scientific inquiry, the results of which add to the scientific body of knowledge. In the context of AI3SD, we have chosen to view scientific discovery as the identification of regular relationships and their underlying causes in a given problem domain by model-based reasoning, and the subsequent verification of them by experimentation, while respecting societal constraints arising from ethics and philosophy.

This perspectives article will provide a summary of the background of the core elements of the Network^+^ (scientific discovery and AI), then detail the formation and current activities of the Network^+^ to date, and finish with some reflections on learning so far and considerations on how best to move forward for the rest of the Network^+^’s time span.

## Scientific Discovery through the Ages

To set the scene for the Network^+^ it is useful to review how the nature of scientific discovery has evolved, at least in the UK, since the enlightenment period^10^, and place in context the potential disruption that AI may bring to the philosophy of science.

Beginning with Bacon, the simple inductivist viewed scientific progress as driven by the collection of data. However, as a description of how scientific progress has actually been made, this does not ring true. As Whewell noted in 1860,^11^^,^^12^ scientific progress does not normally proceed from the unreasoning collection of data to the formation of novel hypotheses. Efficient progress usually requires experimentation to be directed by an understanding of how it would affect our understanding.

Bringing order to the judgements and hypothesis that direct scientific discovery is no easy task. For this reason, philosophers of science have often distinguished between the contexts in which scientific discoveries are made and the context of their justification.^13^, ^14^, ^15^ While justifying a hypothesis is clearly driven by the careful collection, ordering, and analysis of data, the context of discovery has historically been more chaotic.

Our progress in science owes much to the judgements, intuitions, hunches, and creativity of the scientists involved, as well as to their chance encounters, proclivities, values, and social settings. According to Mendeleev, he literally dreamt up the periodic table. The theory of special relativity supposedly came to Einstein as he stared idly from the window of a moving tram. Intuition seems key. As Benjamin^16^ put it in 1934, the “act of intuition … seems to arise most readily when the element of effort and control is conspicuously absent”.

Insights direct investigation. Their genesis offers some explanation of why scientific progress can be so unpredictable.^17^ Insight often precedes any formal justification, so cannot be predicted on the basis of the mass of data with which the scientist is presented.

Early attempts to make use of AI not only in the context of justification but also of discovery (for example, meta-DENDRAL^18^^,^^19^) treated scientific discovery as something that could be formalized; made not only automatic but *algorithmic*. Empirically, however, expert systems still enjoy at best only a very niche use in the laboratory, and this seems to tally with the skepticism of even the most analytic philosophers of science as to whether we could bring any formal order to the context of scientific discovery.

Even Hempel^20^, well known for trying to pin down an inductive “logic of confirmation,” argued that artificial systems were not capable of genuine scientific discovery. For, he argued, real scientific discovery involves the introduction of novel concepts and the formulation of the theoretical principles in which such concepts are embedded. However, classical programming architectures impose such frameworks. So while computers may well exceed us in the pace at which they could test or make use of theories, genuine scientific discovery was beyond them, rendering them, to use a concept introduced in 1962 by Kuhn^17^, unable to shift paradigms.

Hempel's skepticism may finally seem outdated, however. There have recently been huge changes not only to the brute power of computers but also the various techniques that come under the banner of machine learning. The power of automated systems to inspect vast quantities of data along many dimensions allows them to identify novel and surprising correlations, in a process far more akin to Bacon's simple inductivism. Doing so at least puts them in the frame genuinely to augment our own capacity for discovery in Hempel's sense. They can direct us to unnoticed patterns and anomalies that put their use not just within the context of justification but also firmly within that of discovery.

Either way, the AI3SD Network^+^ manifests the importance of AI as a tool of progress both in science and in the philosophies of science and mind. With this in mind, we now reflect on the emergence of the current, third wave of AI in the UK.

## The Emergence and Potential of AI

**Figure 2:**
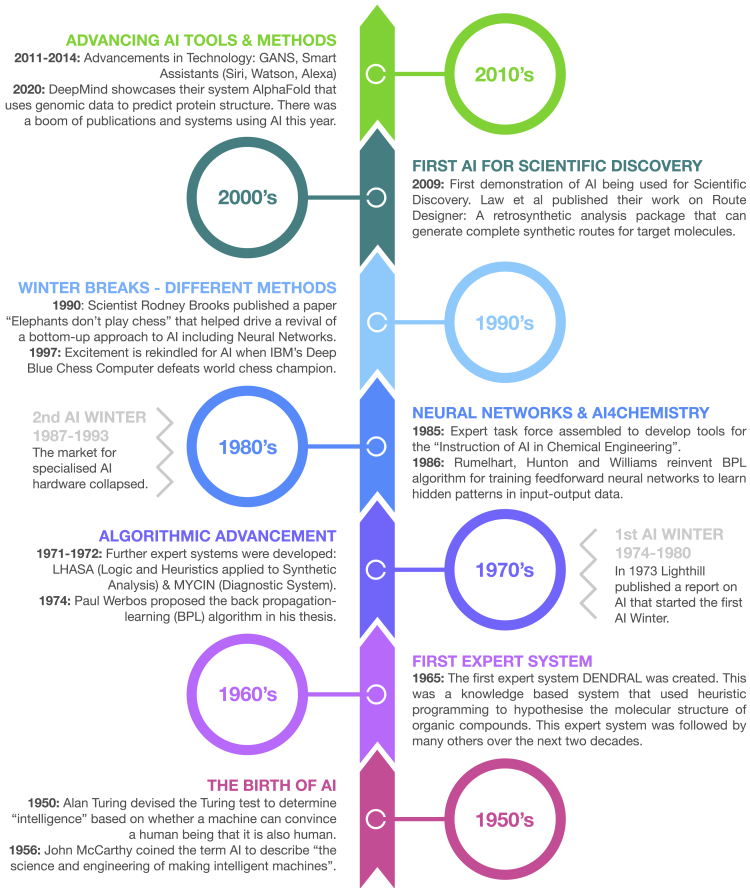
A Timeline of the Development of AI and Its Establishment of a Role in Scientific Discovery from the 1950s to 2020

The history of AI is that of several waves of discovery, followed by hype, followed by winter (as shown in Figure 2).^19^^,^^37^, ^38^, ^39^^,^^24^^,^^25^^,^^21^, ^22^, ^23^, ^42^, ^43^, ^24^, ^44^, ^25^, ^45^, ^46^, ^47^ Although the study of AI was established by the latter part of the twentieth century,^37^ it was hindered by the extravagant claims that had been made regarding the capabilities of digital computers. Both winters were broken by a combination of the advent of new methods and approaches for AI^38^ and the unveiling of exciting new systems using AI,^39^ which reignited the fire and excitement regarding these technologies. However, reviewing these claims, and various of the approaches toward automated discovery, Brannigan concluded in 1989 that the programs then available had not (yet) achieved autonomy in scientific discovery.^40^

At the beginning of the twenty-first century, the UK Government invested in an extensive program of e-science research (often referred to as cyber-science outside the UK, but this generated a different character to the work than the term e-science). In 2009, Hey et al.^41^ edited “The Fourth Paradigm, Data-Intensive Scientific Discovery,” a collection of essays exploring a range of topics in e-science and data science, covering every scientific discipline. In his article, Gray proposed data exploration as the fourth paradigm, following empirical science. theoretical studies, and computational methods, such as simulation.

The UK Government published its own review of the e-science program, also in 2009. References to discovery in this document appear to concentrate on resource discovery (as befits the initial focus of the program on middleware). For example, references to drug discovery resolve to the discovery of information in databases rather than to facilitating the process of actual new molecule discovery. There are but three mentions of machine learning. However, the influence of the e-science program cannot be denied, particularly in changing the way a wide range of researchers work; the UK Computing Research Committee (UKCRC) recommended that computer science research could benefit from using some of the e-science technologies to better facilitate collaboration, sharing test datasets and application repositories (e.g., for machine learning research.” It set the scene for much of the data-driven, cloud-based services-based modern Internet-enabled commerce and research infrastructure. The program set the scene for our modern Internet-enabled and cloud-based commerce and research infrastructure, which is data driven.

As shown by Figure 2, early applications of AI in the chemical sciences were programs for automating aspects of scientific discovery in healthcare and drug discovery (e.g., MYCIN and CADUCEUS)^37^, ^38^, ^39^ or computer-aided design of synthetic routes to unknown molecules (e.g., DENDRAL, CAOS and LHASA).^19^^,^^40^, ^53^, ^54^ The work of E.J. Corey,^44^ which formalized the concept of retrosynthesis analysis, is highly pertinent to computer-aided synthesis. Other attempts have been made, and some are still under active development,^25^ but none would yet challenge a graduate synthetic chemist. Typically, these applications were expert systems using either human-defined rules or rules automatically extracted from databases of organic reactions (with varying degrees of success).

By 2014, significant progress had been made in the area of deep learning,^45^ a methodology that requires the use of enormous amounts of data. Computing power and capabilities had been significantly enhanced such that it was now possible to demonstrate the potential of neural networks, including the use of deep neural networks, which could be trained at scale using graphical processing unit (GPU) computing. Significant advances had also been made with respect to representing unstructured data, such as strings and molecular structures, which could now be represented in a meaningful way to be used in learning algorithms.^46^ At this point in time, AI was seen to have made significant contributions to scientific progress and to have the potential to transform scientific discovery: “However, AI has a far broader capacity to accelerate scientific discovery, and AI-based systems that can represent hypotheses, reason with models of the data, and design hypothesis-driven data collection techniques can reduce the error-prone human bottleneck in scientific discovery.”^47^

In 2011, Kayala and Baldi^48^ presented their work on using neural nets for chemical reactions, with further promising work being conducted in 2017.^49^^,^^50^ Enumerating the multitude of possible routes to a molecule is a straightforward (if large) task, which gives a network connecting available starting materials to the target molecule, with intermediate molecules as the nodes and reactions connecting the nodes. However, to select an optimum route (let alone one that will work) is still a challenge, which needs to be solved to support the growing demand to revolutionize synthetic chemistry to support the green and circular economies. To meet this challenge, AI has to be able to predict the outcome of an unknown reaction.

Artificial and augmented intelligence (AAI) techniques have the potential to significantly surpass current industrial applications^62^, ^63^, ^64^ and, when applied to large-scale heterogeneous data derived from molecular interactions,^65^, ^66^, ^67^ to extract information vital to scientific discovery. As these techniques progress it will be important to be able to understand how AAI has enabled or accelerated a discovery. Further, making use of the philosophical underpinnings of AI and discovery will provide a common language that enables the AI and physical science communities to engage in joint endeavors. The output of these endeavors will directly affect the nature of what discovery and theory will mean in the next decades and the skills set that the AI generation of researchers will need to develop.

## The Network^+^

The Network^+^ was conceived to accelerate scientific discovery using new AAI techniques and to facilitate the use of scientific ideas to enhance the capabilities of AI. The vision and themes of the Network^+^ are outlined here, providing a snapshot of our activities for the first year or so and some thoughts on the future (prior to the global lockdown, which, of course, is leading us to rethink the nature of our networking activities and the focus of research areas).

### The Network^+^ Vision

We are living through a data revolution, which will be as transformative of our society as the industrial revolution. Algorithms, and in particular learning algorithms, are the engines of this revolution. So-called intelligent algorithmic systems affect many areas of our personal and professional lives, making decisions based on prior “learned” knowledge. The use of learning algorithms has the potential to revolutionize the way science develops. The Network^+^ is focused on training researchers to develop and deploy AAI for scientific discovery in the chemical and material sciences, in order to truly realize this potential.

The timeliness of the Network^+^ is highlighted by the fact that the current volume of chemical reaction data is far beyond what humans can effectively use (but, as we will see, accessible and useful data are a very limited and valuable resource). Murray et al.^57^ have estimated that, for the specific case of a typical transition-metal-catalyzed reaction, there are over 50 million applicable reaction permutations. At 10^7^, this assessment is small compared with the enumeration of chemical space. Reymond^58^ has generated a database, GDB-17, of molecules of up to 17 atoms of C, N, O, S, and halogens, yet, at 10^11^, this comprises a mere fraction of the estimated 10^60^ molecules obeying Lipinski's rule-of-five, which was developed to aid the identification of potential drug candidates by their oral bioavailability.^59^ Even higher estimates, up to 10^180^, can found in the table published by Gorse,^60^ although the more commonly accepted number is in the region of 10^80^.

Furthermore, we are at the start of an explosion in the availability of detailed reaction data due to the increase in this data being recorded digitally (e.g., the use of electronic laboratory notebooks) and the use of robotic reaction systems, which improve data repeatability and increase throughput. Supporting these capabilities are advances in high-throughput analytical methods. Automation has for a long time played a significant role in improving data collection. With our ability now to generate or access huge datasets, machine learning techniques are playing an ever more significant role in data analysis. Finally, with the emergence of sophisticated deep learning systems designed to simulate aspects of human neurological function, we may be on the verge of augmenting or automating some aspects of the creative skills that drive scientific progress.

Impressive developments have recently taken place around deep neural network architectures. These are layered networks with trainable parameters forming complex non-linear mappings between input and output data. Learning of such mappings has the property of extracting useful latent patterns in complex high-dimensional spaces. Although, over past decades, it was thought sufficient to work with layered networks that were shallow (i.e., one hidden layer between input and output), and estimating parameters of networks with deep architectures was considered technically difficult, recent advances along several lines of architecture specification and optimization tricks have resulted in such powerful architectures, demonstrating impressive empirical results in diverse domains such as computer vision, automatic speech recognition, machine translation, and computational biology, and playing challenging games such as chess and Go. Learning of latent representations and making accurate predictions endow such models with the power to augment the role of scientists by reducing the space in which one might search for hypotheses or in the ranking of hypotheses in terms of some past experience extractable from data.

So, computing techniques, particularly machine learning algorithms, are no longer there solely to increase the number of tasks a scientist can complete; they now stand ready to alter the role of the scientist. The vision of AI3SD is to bring together researchers from both science and technology backgrounds to show how cutting-edge artificial and augmented intelligence technologies can be used to push the boundaries of scientific discovery.

Our core aims are to:

• Make use of AAI to drive scientific discovery.
• Use the needs of scientific discovery to drive new techniques in AAI.
• Provide new and innovative applications to the chemicals and materials sectors.
• Address ethical and philosophical issues associated with AAI in an informed manner to ensure adherence to the best possible ethical practices.
• Train researchers to create and use new systems of AAI to push the limits of the synthesis of molecules and the engineering of materials properties, enabling discovery and exploitation of unseen, unexplored, and currently unimagined parts of chemical and material space.

### Network^+^ Landscape and Themes

We chose the design and synthesis of chemicals and materials (i.e., property prediction, synthesis prediction, and materials and device manufacture) as the inter-related areas for the initial cutting-edge exemplars on which to focus the landscape for the Network^+^’s activities. Molecular compounds underpin just about every aspect of our lives, from sustainable energy, to construction, to healthcare. Society's demands for enhanced performance is far outweighing the capability of the research community to discover materials that deliver it. Furthermore, an inability to then rapidly transform new compound discoveries into usable functional materials exacerbates the shortfall. Such discoveries are critical to meeting the majority of the UN sustainability goals,^61^ and are at the top of the UK industrial strategy.^62^

The complexity of the relationships between chemical/molecular structure, physical properties, and material (and device) performance renders the understanding and prediction of these relationships for the most part intractable by conventional approaches to computation. That the area is ripe for exploitation in the UK is highlighted by the involvement of big and innovative companies, as well as start-ups (e.g., at least 47 start-ups in the area of AI and drug discovery, as of 2018,^63^ based in the UK).

In following these general directions, the main themes that have evolved in the first year of the Network^+^ are:

• Novel machine learning for chemical and materials discovery
• Novel active learning AI in the chemical space
• Novel aspects of AI and drug discovery
• New AI-based methods (and methodologies) for scientific discovery

### Forming the Network^+^

In establishing the Network^+^, we were able to draw on our valuable previous experience with the networks and challenge areas described in the Past Experiences box. The lessons learned from the ITaaU Network^+^ were particularly useful with regard to organizational matters, such as the importance of creating resources derived from workshops, funded projects, and conferences, and how to run different types of meetings in the most effective manner.^75^, ^76^, ^77^

### Previous Experience

**Past Experiences: Why We Wanted to Run the Network**

The investigators have had extensive experience in running and working with networks and collaborating on projects across different areas of AI and chemistry, all of which fed into how we formed and run the Network^+^. Some of the main projects and networks with which we have been involved are listed below:

Networks, centers, services, and Projects:

• ITaaU: http://www.itutility.ac.uk/
• Dial-a-Molecule Grand Challenge Network: http://generic.wordpress.soton.ac.uk/dial-a-molecule/
• Digital Economy Network: https://digitaleconomynetwork.com/
• Leverhulme Research Centre for Functional Materials Design: https://www.liverpool.ac.uk/leverhulme-research-centre/
• UK National Crystallography Service: http://www.ncs.ac.uk/
• CombeChem - http://www.combechem.org/

We are distilling our main experiences in running networks and will present online resources in the near future (look for How to Train your Network).

For the scientific research perspective, we were able to rely on our formative experience with the CombeChem science project,^67^ and our extensive combined background in machine learning, chemistry, philosophy, and working in interdisciplinary projects that spanned these areas.

The University of Southampton has maintained a strong focus on digital chemistry^68^ since the late twentieth century, which has extended into smarter ways of conducting science,^80^, ^81^, ^82^, ^83^ and digitizing and managing scientific research,^84^, ^85^, ^86^ and also the means whereby scientific methodology can be applied in broader areas, such as for the ITaaU Network^+^. In chemistry, this has involved research into the impact of digital technology on area such crystallography, crystal structure prediction, combinatory chemistry, materials discovery, molecular simulations, chemical modeling, and drug discovery.

As our interest in digital chemistry has evolved, we have become increasingly aware of the potential of AI. Research in AI at the University of Southampton spans a range of topics in machine learning, agent-based modeling and mechanism design, Semantic Web technologies,^76^ and Web science. Where machine learning augments scientific discovery, a likely formulation of data-driven modeling is that of detecting outliers in data. Expertise closely related to this problem, the purpose of which is the use of models to circumvent the curse of dimensionality encountered in probability density modeling, is in the areas of regression^77^ and structured matrix methods,^78^ which have been applied in our recent work to detect cellular protein regulation,^77^^,^^79^^,^^80^ low-dimensional representations of protein sequences,^81^ and in vaccinology.^82^

Other aspects of AI have also been studied by Southampton philosophers, who have expertise in the epistemology, logic, and ethics of AI.^83^ The question of the interpretability of artificial neural networks is explored from an epistemological perspective, and held up as a model against which to better understand the epistemology of natural perceptual systems.^84^ Meanwhile, the possibility that the reliability profiles of such systems might remain inexplicable has pressing ethical implications, which it is argued cannot be ignored. The philosophy department has had close ties with several ongoing projects in law, politics, engineering, and computer science, including playing advisory roles for both AI3SD and the Web Science Institute. These ties were strengthened by an international conference on artificial ethics in 2017, co-organized with Southampton's Autonomous Systems Unmanned System Research Group (USRG). Further, AI3SD recently ran a discussion-based workshop on the Ethical and Societal Implications of using AI for Scientific Discovery, a full report of which is now available.^85^

This combined experience of the Network^+^ team has proved very valuable, ensuring that we have adequate expertise for the interdisciplinary nature of using AAI to accelerate scientific discovery.

### Network^+^ Membership

Membership of the Network^+^ is defined by our mailing list, and in the first year we have rapidly established a wide-ranging interdisciplinary community that not only brings together researchers from the physical (and life) sciences and computer sciences but also across both academia and industry. As our network is funded by UKRI, it is primarily UK based, although we do have members from outside the UK. As of 22/09/2020 we have over 840 members, with representatives from over 60 different universities and over 80 different companies. The Network^+^ also has a strong social media presence, with over 470 twitter followers (@AISciNet), and an increasing following on LinkedIn.

### Network^+^ Events and Activities

At AI3SD we aim to bring together researchers looking to show how cutting-edge artificial and augmented intelligence can be used to push the boundaries of scientific discovery. As such, an important objective for the Network^+^ was to establish a portfolio of activities and support for early-stage research. Since its launch in December 2018, AI3SD has organized or co-organized 12 events. These events comprised five main types: workshops, conferences, training events, hackathons ,and town meetings (for funding). A comprehensive list of the events that have been organized or co-organized by AI3SD can be found on the AI3SD events page.^86^

#### Workshops

Our workshops focus on a particular topic, with the structure typically comprising an introduction to AI3SD aims for the themes of the day, followed by keynote talks from experts in the field, working group discussions, and concluding with summing-up exchanges of views. The time allocation varies according to the specific topic and the aims of the workshop, in some cases emphasizing networking, while others give priority to identifying key issues and emergent themes. Since the launch of the Network^+^, there have been seven workshops, covering the key themes of the Network^+^ (drug discovery; molecules and materials; chemical design and discovery). Each workshop has been formally recorded with a professional report^87^ published on the Network^+^ Web site and with a formal DOI assigned.

#### Conferences

These were either single- or multi-day meetings that aimed to bring together all members of the Network^+^ to learn about and discuss a range of relevant themes. These conferences took similar forms to the workshops in that they comprised presentations with some discussion sessions (although typically there were many more presentations), and sometimes had short workshops embedded as part of the program. The presentations were on a wider range of topics than for just a workshop, as these conferences were aimed at the Network^+^ as a whole and looked to cover a wide range of relevant themes to appeal to a much larger audience. AI3SD has held two major conferences so far, the launch event in December 2018 and the big annual conference in November 2019. Both of these have also been formally recorded with a report.^87^

#### Training Sessions

Two training events have been run thus far as part of AI3SD. One was a funding workshop that was run as part of the second town meeting. This provided tips on writing funding applications, IP for AI, and writing sustainable research software. The second was a training session on machine learning and data science for chemistry, which comprised training presentations on these different areas, and training on a number of different datasets that were going to be used for a hackathon.

#### Hackathons

These hack sessions were hosted to allow Network^+^ members to encourage creativity and provide time to try out new methods in a supportive environment with experts on hand to help.

#### Town Meetings (for Funding Calls)

These events were hosted to explain the processes of the AI3SD funding calls, to provide prospective candidates with enough information about how to apply, and also served as a place for Network^+^ members to network and potentially find new collaborators. These meetings were not formally recorded in a report, but the questions and answers given were all recorded and put on the Funding FAQ page^88^ to serve as a useful resource for applicants.

### Project Funding Mechanism

Part of being a Network^+^ is the inclusion of a special budget to run our own funding calls to support some short £50,000 pilot projects. Two funding calls were run, which led to supporting seven projects overall; details (and available reports) can be found on the AI3SD Funded Projects page.^89^ These funding calls were extremely competitive, with 50 separate applications being sent in over the two funding calls, including 63 different universities, other academic institutions, and companies, and 138 individual people. Each proposal was reviewed by at least two members of the Advisory Board and, in both rounds, one person read all of the proposals to help calibrate the variation among reviewers. A key aspect that we were looking for in these applications was projects that could demonstrate long-term (as well as short-term) pathways to impact such that these projects could keep moving forward past the initial pilot project period, thus making the best use of this funding allocation.

## Reflections and Going Forward

This iteration of the Network^+^ is set to run until June 2021, and, at this halfway point, it is important to evaluate lessons learned so far and identify the main challenges that we have identified during the research that has been undertaken.

### What Have We Learned so far?

There are three clear lessons that we have learned so far:

(1) First, the importance of the interdisciplinary approach for accelerating scientific discovery through AAI; it is vital to include experts in the many different areas of AAI, chemistry, philosophy, and ethics to identify the range of challenges from these different disciplines and to provide different approaches and skills to solving problems.
(2) Second, the combination of academic and industrial expertise has been an imperative as these bring different techniques, priorities, and access to resources/data that, when combined, present brand new opportunities. Industry is ahead of academia in many areas, and enabling access by academics to the large amounts of data held in industry can prove incredibly effective, as demonstrated by the recent coronavirus disease 2019 (COVID-19) collaborations.
(3) Third, from the presentations and discussions that we have engaged with to date, we have identified key challenges in the chemical and AAI spaces that are detailed later.

### Impacts

Thus far, our short-term impacts have been realized in three main areas. Our growing membership; this is defined by our mailing list, which now has over 840 members and is continuing to grow at a steady pace. Second are the outputs of our workshops, conferences, and funded projects (see AI3SD Reports page^87^), which have led to the identification of the main challenges in the Network^+^ research space (detailed below). Our third main impact has been the collaborations that have formed due to this network: both between AI3SD and other parties and between other academic/industry bodies either due to collaborating for AI3SD funding calls or from seeking out potential collaborators from the AI3SD Network^+^. Our plans for longer-term impact are detailed later.

### What Challenges Have Been Identified from the Network^+^ Research Thus far?

A significant output of the events and funding calls run thus far by the Network^+^ (in addition to the individual research undertaken by the Network^+^) has been the identification of the main discovery challenges in the chemical and AAI spaces. Further, this has highlighted the importance of interdisciplinary training as an important and worthwhile issue, as many of these challenges require significant knowledge in more than one domain.

### Chemical Space Challenges

• Recognition of the extent of chemical space and other data spaces.
• Heterogeneity of data spaces and the ability to link data (also mashups).
• Creating greater quantities of high-quality data, with the emphasis on quality.
• How to exploit unseen, unexplored, and currently unimagined parts of chemical and material space.

### AAI Space Challenges

• Understanding the new pathways to discovery opened by the application of AI
• How to involve a wider range of the computer science community
• Engage the mathematics community
• Understanding how to ensure that AAI for scientific discovery is both ethical and explainable

### Future Plans: 2020 and Beyond

There are several different threads of future plans for AI3SD: running workshops and conferences, creating useful resources for the AAI for scientific discovery community, working with the recipients of the AI3SD Funded Pilot projects to help them realize their impact, and working to make the Network^+^ sustainable for the future.

As an interim measure to mitigate the impact of COVID-19, we moved our activities online for the rest of 2020 with the launch of our online summer seminar series, and are continuing to engage regularly with our members through newsletters and our mailing list, which is continuing to grow at a steady pace despite the pandemic. We are currently planning for 2021 with strategies for either continuing online or slowly progressing back to face-to-face meetings depending on how the COVID-19 pandemic progresses.

Having explored the different areas of each of the Network^+^ themes and identified some key overall challenges, the next steps are to delve further into these spaces. Data and all the aspects characterizing it (availability, quality, metadata, linking potential) have been identified as a key area for AAI for scientific discovery (and indeed any AI/machine learning endeavor). Further interdisciplinary workshops and hackathons will be run in this area, aiming to provide training in understanding and adhering to different data standards and best practices across different disciplines with respect to data governance and data publishing. There will also be training on different techniques for text mining, data linking, and creating or converting data to be AAI ready. In particular, a dual datathon and hackathon will be run, allowing participants to attend either or both, whereby data are cleaned, linked, and analyzed in the first instance, and then used with AAI techniques following this. Further, additional interdisciplinary workshops will also be run in the areas of symbolic AI and machine learning for small deep learning, statistical mechanics and machine learning, AI and molecular dynamics, and AI/machine learning/science and casual reasoning.

AI3SD has started to expand its remit to creating and linking to useful resources for the Network^+^ members to use. The AI3SD Resources page^90^ now links to resources for data, learning, literature, and software for AAI for scientific discovery; resources for these sections are being researched currently and the pages are incrementally updated. There is also a resources section for our COVID-19 researchers where datasets, software, and online platforms are being collated to help Network^+^ members. Further, the Network^+^ will be undertaking some horizon/literature scanning exercises in the challenge spaces that have been identified.

Working with the recipients of the AI3SD Funding Awards will enable both them and the Network^+^ to make further impact. As noted above, a key assessment criterion for selecting projects to fund was based on the potential for long-term impact as well as short and medium term. The Network^+^ Team (including our Advisory Board with a wide variety of expertise) will continue to work with the recipients of the funding to see how their work can be taken forward and where further funding can be applied for. Due to the impacts of COVID-19, we have granted each funded project a no-costs extension, and are working with the principal investigators of each project to ensure that they receive the time and help they need to finish their projects before this term of the Network^+^ ends.

Finally, a key area for future planning is to work toward making the Network^+^ sustainable such that it can continue as a self-sufficient body once the main funding term has finished.

### Resource Availability

#### Lead Contact

Further information and requests for resources should be directed to and will be fulfilled by the lead contact, Samantha Kanza (s.kanza@soton.ac.uk).

#### Materials Availability

A wide range of information, including all of our conference and event reports, funding reports, and details about the activities of the Network^+^ can be found on our website (www.ai3sd.org).

#### Data and Code Availability

Not available.
